# Poor Boys

**Published:** 1873-04

**Authors:** 


### POOR BOYS.

The ranks of the rich and great, and famous and good, are constantly and mainly filled up by those who were once friendless and poor.

Peter Cooper, when a boy, worked for twenty-five dollars a year.

Horace Greeley worked for forty dollars a year, and saved the whole amount to be sent home to help his father and mother.

Governor Palmer, of Illinois, was once a blacksmith "very fine."

A little one-eyed French boy began life by bottling cider, made millions, and founded Girard College, which takes up a hundred little, ragged, orphaned boys from the streets and gutters and cellars of Philadelphia every year; feeds, clothes, educates, and sends them out into the world, in due time, good mechanics.

A. T. Stewart began life in New York with a good education and a thousand dollars in money.

Cornelius Vanderbilt earned his first shillings by rowing travelers across the Hudson, between New York and Jersey City, at twelve and a half cents each.

